# Acute and Chronic Immunological Responses to Different Exercise Modalities: A Narrative Review

**DOI:** 10.3390/healthcare13172244

**Published:** 2025-09-08

**Authors:** Ebru Sever, Sıla Yılmaz, Mitat Koz

**Affiliations:** 1 Physiotherapy and Rehabilitation Doctorate Program, Graduate School of Health Sciences, Istanbul Medipol University, Istanbul 34815, Türkiye; ebru.sever@std.medipol.edu.tr; 2 Department of Physiotherapy and Rehabilitation, Faculty of Health Sciences, Kocaeli Health and Technology University, Kocaeli 41050, Türkiye; 3 Department of Physiotherapy and Rehabilitation, School of Health Sciences, Istanbul Medipol University, Istanbul 34815, Türkiye; 4 Department of Exercise and Sports Sciences, Faculty of Health Sciences, Eastern Mediterranean University, Gazimagusa 99628, Cyprus; mitat.koz@emu.edu.tr

**Keywords:** cytokines, exercise, inflammation mediators, immune system, lymphocytes, physical activity

## Abstract

The relationship between exercise and immune function has been widely studied, yet findings remain inconsistent regarding how different exercise modalities and intensities influence acute and chronic immunological responses. Previous reviews have often focused on single exercise types or limited outcomes, leaving a gap for an integrated synthesis. This narrative review aims to address this gap by summarizing and comparing immunological effects across aerobic exercise, resistance training, high-intensity interval training (HIIT), blood flow restriction (BFR), isometric exercise, mind–body interventions, and hypoxic training. A structured narrative approach was adopted. Literature published between January 2000 and December 2024 was searched in PubMed, Scopus, and Web of Science. Experimental and observational studies on humans and animal models were included, with study selection and data extraction performed by two reviewers. Findings were synthesized thematically by exercise modality to capture both acute and chronic immune responses. Twenty-four eligible studies were identified. Aerobic and mind–body exercises consistently demonstrated anti-inflammatory and immunoprotective effects, including increased IL-10 production, improved T cell profiles, and reduced inflammatory markers. Isometric training showed favorable modulation of cytokines and T cell balance, while resistance training evidence was limited but suggested cortisol-lowering benefits. HIIT, BFR, and hypoxic exercise produced mixed results, often combining transient pro-inflammatory responses with immunological benefits. Acute and chronic immunological responses to exercise are highly modality- and intensity-dependent. Aerobic and mind–body interventions provide the most consistent benefits, whereas HIIT, BFR, and hypoxic training show variable effects. Further high-quality trials are needed to clarify mechanisms and guide exercise-based immune recommendations.

## 1. Introduction

Exercise has long been recognized as a modulator of immune function, yet findings remain inconsistent and often contradictory. Early studies in older adults demonstrated both beneficial and suppressive effects depending on exercise characteristics [1]. However, later studies revealed a more complex picture, as findings indicating that moderate-intensity exercise is frequently associated with anti-inflammatory and immunoprotective benefits, prolonged or excessive exercise may suppress immune function [2]. These inconsistencies have limited the development of clear, evidence-based recommendations for both clinical practice and athletic training. Thus, a more integrated understanding of how exercise influences immune function is required. To address this need, the present review provides a broad synthesis of current knowledge on exercise and immunity.

Understanding the immune consequences of exercise has broad clinical and societal implications, as the immune system plays a fundamental role beyond host defence. It maintains tissue homeostasis and integrity, and interacts closely with key physiological systems such as metabolism, the central nervous system, and the cardiovascular system, thereby contributing to the prevention of chronic diseases, healthy aging, and performance optimization [3]. The importance of these functions is evident across different populations. Evidence from elderly women shows that moderate cardiorespiratory exercise reduced upper respiratory infection incidence, suggesting a protective effect of regular activity in aging populations [1]. In athletic populations, acute respiratory infections are among the most common causes of impaired performance at major competitions, and both athletes and coaches widely perceive that heavy training loads predispose to upper respiratory tract infections, with many elite athletes reporting episodes that interfered with training and competition [4]. Beyond athletic settings, evidence from large-scale epidemiological studies shows that in the general population, engaging regularly in moderate to vigorous physical activity is associated with 31% lower risk of community-acquired infectious disease and 37% lower risk of infection-related mortality [5].

Despite decades of research, it remains unclear how different types, intensities, and durations of exercise distinctly influence acute and chronic immunological outcomes. This raises a central research question: how do specific exercise modalities shape immune cell responses, inflammatory mediators, and overall host defense across populations? To address this question, the present narrative review systematically synthesizes evidence published between January 2000 and December 2024, focusing on aerobic, resistance, high-intensity interval training (HIIT), blood flow restriction (BFR), isometric exercise, mind–body practices, and hypoxic training. By comparing findings across these modalities, this review aims to provide an integrated perspective on exercise-induced immunological responses, highlight consistent patterns and discrepancies, and outline future directions to guide both clinical practice and athletic performance.

## 2. Materials and Methods

This structured narrative review adopts selected methodological principles from structured reviews, including predefined eligibility criteria, comprehensive database search strategies, and thematic synthesis of findings.

### 2.1. Research Strategy

This narrative review examines the effects of different exercise modalities on immune function by synthesizing findings from original research studies and review articles published between January 2000 and December 2024. This timeframe was chosen to enhance methodological consistency and comparability, as standardized immunological assays and the investigation of modern exercise modalities have become widespread since 2000. Earlier studies were excluded because they often relied on heterogeneous laboratory methods and rarely addressed these modalities. The literature search was conducted in PubMed, Scopus, and Web of Science, which together provide comprehensive and complementary coverage of biomedical and multidisciplinary research. These databases were selected to maximize sensitivity and specificity while avoiding unnecessary duplication across sources. The search strategy combined controlled vocabulary (MeSH terms) and free-text keywords. MeSH terms included “immune system”, and “exercise intensity”. Additional keywords such as “exercise immunology”, “exercise type”, and “exercise duration” were incorporated to capture studies not indexed under MeSH. The search strategy was developed using combinations of the following keywords: “immune system AND exercise immunology”, “exercise type AND immune system”, “exercise intensity AND immune system”, and “exercise duration AND immune system”. In all three databases, filters for publication type (original research and reviews) and language (English or Turkish) were applied at the search stage, in line with the predefined eligibility criteria. Additional exclusions (e.g., case reports, conference abstracts, theses, dissertations, editorials) were implemented during the screening process. To minimize bias, database searches and study selection were performed independently by two authors, and disagreements were resolved through discussion.

### 2.2. Eligibility Criteria

To ensure the relevance and timeliness of the data included in this review, clearly predefined eligibility criteria were established.

Studies were included if they met all of the following criteria:

Published between January 2000 and December 2024,

Experimental, observational review in design (including systematic reviews, meta-analyses, and narrative reviews),

Conducted on human or animal models,

Investigated at least one parameter related to exercise (type, intensity, or duration) and its effect on immune outcomes,

Full text available,

Published in English or Turkish.

Studies were excluded if they met any of the following criteria:

Duplicate publications,

Case reports, conference abstracts, theses, dissertations, and editorials,

Articles without a clearly described methodology or reported results,

Studies that did not assess immunological biomarkers or cellular immune parameters.

**Figure 1:**
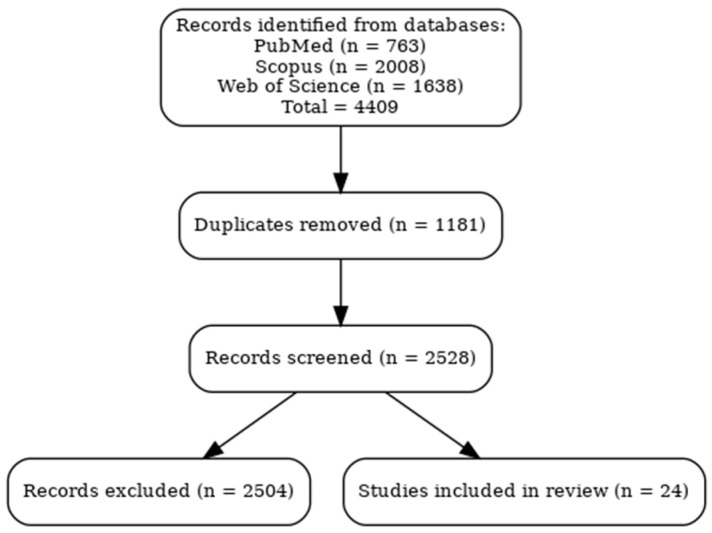
PRISMA flow diagram.

In addition to original research, selected high-quality narrative and systematic reviews were included to provide mechanistic insights and contextual understanding where relevant. In total, database searches yielded 763 records from PubMed, 2008 from Scopus, and 1638 from Web of Science, giving 4409 records after merging. After removing 1181 duplicates and excluding 2504 records (editorials, case reports, abstracts, and studies not meeting the inclusion criteria or meeting exclusion criteria, such as those studies without immune outcomes), 24 articles met the inclusion criteria and were analyzed in this review. The study selection process is summarized in a PRISMA flow diagram (Figure 1).

The extracted data were synthesized thematically based on exercise type (aerobic, resistance, HIIT, BFR, isometric, hypoxic, and mind–body interventions). Within each modality, findings were further organized by type of immune outcome (e.g., cytokine responses, immune cell subsets, hormonal markers) and by acute versus chronic responses where available. Both consistent and contradictory findings were extracted and reported, and possible explanations for discrepancies (e.g., methodological differences, participant characteristics, intervention protocols) were considered in the Discussion.

## 3. Results

Database searches initially identified 4409 records. After removing 1181 duplicates and excluding 2504 records based on eligibility criteria, 24 studies were included and analyzed. The study selection process is illustrated in Figure 1 (PRISMA flow diagram). Both acute and chronic immune responses were considered where data were available. Findings are presented under thematic categories, comparing exercise modalities—BFR training, mind–body interventions, hypoxic training, isometric exercise, aerobic exercise, resistance exercise, and HIIT—and their reported effects on immune function. Within each category, results are summarized according to key immune outcomes and study design features.

Table 1 provides a summary of the four studies that met the eligibility criteria and examined the effects of BFR training on immune function. Based on the results of these studies, the immune effects of BFR training appear to be variable and largely dependent on exercise intensity, duration, and population characteristics. Across the four studies included in this review, acute BFR exercise was associated with elevations in muscle damage markers, such as creatine kinase, particularly during high-intensity protocols. For instance, Neto et al. (2018) reported significant protocol × time effects, with CK increasing by ~178% at 24 h (*p* < 0.001) and remaining elevated at 48 h (*p* = 0.002) in the high-intensity resistance group, whereas both continuous and intermittent low-load BFR protocols showed smaller increases that remained significantly lower (*p* = 0.035–0.049) [6]. Shimizu et al. (2016) reported increased plasma norepinephrine, lactate, vascular endothelial growth factor, and growth hormone responses following low-intensity BFR sessions, suggesting an acute stress and hormonal activation effect (each *p* < 0.01) [7]. At the cellular level, Nielsen et al. (2017) demonstrated that BFR training influences macrophage polarization, promoting both pro-inflammatory (M1; *p* < 0.05) and anti-inflammatory (M2; *p* < 0.01) phenotypes in skeletal muscle tissue [8]. These findings indicate that BFR may initially trigger transient inflammatory and stress-related responses, which could potentially contribute to muscle adaptation over time. Additionally, Behringer et al. (2017) demonstrated that moderate-intensity eccentric leg press with BFR induced marked muscle swelling, metabolic stress, and acute elevations in growth hormone, indicating possible hormonal mechanisms contributing to muscle adaptation [9]. Taken together, these findings suggest that BFR training elicits acute stress and inflammatory responses, but the magnitude and direction of immune effects vary with intensity, duration, and population characteristics.

Five studies met the eligibility criteria and investigated the impact of mind–body approaches, including cognitive behavioral stress management, meditation, yoga, and rhythmic breathing techniques, on immune parameters (Table 1). Evidence from these studies, mainly conducted in populations with chronic health conditions such as Human Immunodeficiency Virus (HIV) infection and cancer, demonstrated positive immunomodulatory effects. Reported outcomes included significant increases in CD4+ and CD8+ T-lymphocyte counts at one-year follow-up in the cognitive behavioral stress management group compared to controls (F(1,22) = 6.01, *p* < 0.03; F(1,22) = 6.40, *p* = 0.019), along with enhanced NK-cell activity, improved IgG titers, and reductions in stress-related hormones such as cortisol (b = –0.25, *p* < 0.09) and norepinephrine [10,11]. Similarly, in melanoma patients, structured psychiatric group interventions were associated with elevated lymphocyte counts and NK cell cytotoxicity, alongside a slight decrease in CD4+ T cell percentages at the 6-month follow-up [12]. Kochupillai et al. further reported that rhythmic breathing techniques increased NK cell counts in cancer patients, although no significant changes were observed in T cell subsets [13]. Additionally, Tooley et al. reported that both TM-Sidhi and other yoga-based meditation practices were associated with increased melatonin secretion, with post-treatment melatonin levels significantly higher on meditation nights compared to control (TM-Sidhi: F(1,9) = 11.12, *p* < 0.01; Yoga: F(1,6) = 16.19, *p* < 0.01), suggesting possible neuroendocrine-immune interactions [14]. Overall, these findings indicate that mind–body interventions can beneficially modulate both cellular and hormonal aspects of immunity, though effects may differ by intervention type and clinical population.

Five studies were included to evaluate the impact of hypoxic exercise on immune parameters (Table 1). Evidence from these studies suggests that hypoxic exercise exerts mixed effects on immune parameters, largely dependent on exercise intensity, exposure duration, and study population. Meta-analytic findings reported a greater increase in the anti-inflammatory cytokine IL-10 under hypoxic compared to normoxic conditions (SMD = 0.60, 95% CI 0.17–1.03, *p* = 0.006), accompanied by elevated TNF-α levels in some protocols (SMD = 0.17, 95% CI −0.10 to 0.46, *p* = 0.21) [15]. Individual trials demonstrated heterogeneous outcomes; for example, one study found broad increases in IL-6, TNF-α, IL-1ra and IL-10 immediately post-exercise (all *p* < 0.05) [16], whereas another reported a more selective response, with IL-6 elevated post-exercise and after 2 h recovery compared with baseline and pre-exercise values (*p* < 0.05) [17]. Animal models indicated mucosal inflammation and increased circulating pro-inflammatory cytokines after normobaric hypoxia exposure [18]. Narrative review evidence also suggests a potential shift toward anti-inflammatory responses, although findings were not uniform across all cytokines assessed [19]. Taken together, these results suggest that hypoxic exercise elicits both pro- and anti-inflammatory responses, with outcomes varying by protocol and population, underscoring the need for further research to clarify its net immunological effects.

Four studies that met the eligibility criteria were analyzed to assess the effects of isometric exercise on immune markers (Table 1). These studies demonstrated beneficial effects on specific immune parameters, though findings varied across populations and protocols. In prehypertensive adults, Ogbutor et al. reported that isometric handgrip training increased IL-10 levels, improved the CD4/CD8 T cell ratio (*p* < 0.05), and reduced both TNF-α and IL-6 concentrations, alongside a decrease in CD8 counts, indicating favorable modulation of adaptive and anti-inflammatory immune responses [20,21]. In elderly women with early knee osteoarthritis, Park et al. found that combining isometric exercise with whole-body electromyostimulation reduced circulating inflammatory markers, including IL-6, TNF-α, CRP, and resistin, while improving muscle strength and functional performance (*p* < 0.001 for all) [22]. Conversely, a quasi-experimental study in sedentary obese women reported no significant changes in most leukocytes, including neutrophil, lymphocyte, monocyte, or eosinophil counts, except for a difference in basophil levels, suggesting that traditional resistance training and total body resistance exercise protocols may have limited effects on broader leukocyte populations in certain conditions [23]. Overall, these findings indicate that isometric exercise can beneficially modulate inflammatory and adaptive immune responses, though effects appear to depend on health status and exercise protocol.

Three studies investigating the effects of aerobic exercise on immune parameters were included in the review (Table 1). Abd El-Kader et al. reported that 6 months of aerobic training in sedentary elderly adults significantly increased anti-inflammatory cytokine IL-10 (*p* < 0.05) and T cell subsets (CD3+, CD4+, CD8+; all *p* < 0.05), while reducing IL-6, TNF-α and adhesion molecules (ICAM-1, VCAM-1, E-selectin; all *p* < 0.05). Aerobic exercise produced greater improvements than resistance training, including a beneficial reduction in the CD4/CD8 ratio (*p* < 0.05) [24,25]. Similarly, in patients with chronic obstructive pulmonary disease, Abd El-Kader et al. (2016) found that 12 weeks of aerobic exercise significantly decreased TNF-α, IL-2, IL-4, IL-6, and CRP levels, with reductions being significantly greater than those observed with resistance training (*p* < 0.05) [26]. Overall, these findings suggest that aerobic exercise exerts consistent anti-inflammatory and immunoenhancing effects, often surpassing those of resistance training.

Only one study met the eligibility criteria for isolated resistance training protocols and included in the review to evaluate the effects on immune parameters (Table 1). Taha & Mounir demonstrated that low- to moderate-intensity resistance exercise (30–50% 1RM) significantly reduced serum cortisol levels (both *p* = 0.001), whereas high-intensity exercise (80% 1RM) did not produce a significant change (*p* = 0.144) in elderly participants [27]. These findings suggest that resistance exercise may exert beneficial endocrine effects primarily at lower intensities, although evidence remains limited to a single study and warrants further confirmation.

Two studies met the eligibility criteria and were analyzed to assess the effects of HIIT on immune parameters (Table 1). Paolucci et al. observed that HIIT significantly increased IL-6 compared to both moderate-intensity continuous training (*p* = 0.04) and controls (*p* = 0.02), while TNF-α decreased following moderate-intensity continuous training compared to controls (*p* = 0.01), indicating a more pro-inflammatory profile for HIIT in university students [28]. In contrast, Bartlett et al. reported that both HIIT and moderate-intensity continuous training significantly enhanced neutrophil phagocytosis (*p* = 0.023; *p* = 0.049) and oxidative burst (*p* = 0.030; *p* = 0.004), as well as monocyte phagocytosis (*p* = 0.005; *p* = 0.002) and the percentage of monocytes producing an oxidative burst (*p* = 0.030; *p* = 0.006), indicating improved innate immune defense regardless of exercise intensity [29]. Together, these studies highlight a dual pattern: while HIIT may promote a more pro-inflammatory cytokine profile, both HIIT and moderate-intensity continuous training appear equally effective in enhancing innate immune cell functions, underscoring the complexity of exercise–immune interactions.

Overall, findings across modalities indicate that aerobic and mind–body interventions most consistently promoted anti-inflammatory and adaptive immune responses, whereas BFR and hypoxic training produced more variable or context-dependent effects. Isometric protocols generally improved selected immune and functional markers, while HIIT showed both pro-inflammatory cytokine elevations and enhancements in innate immune cell function. These patterns highlight that exercise modality, intensity, and participant characteristics are critical determinants of immunological outcomes.

Although database searches initially yielded 4409 records, strict eligibility criteria (requiring direct measurement of immune biomarkers in relation to exercise modalities) narrowed the pool to 24 studies. This rigorous selection aimed to ensure methodological consistency and clinical relevance of the evidence synthesized. Furthermore, it should be noted that certain modalities, such as HIIT and isolated resistance exercise, were represented by only a small number of studies, which limits the strength of conclusions that can be drawn for these categories. Table 1 summarizes the characteristics of the studies.

**Table 1:** Study characteristics.

Title, First Author, and Date	Study Design	Sample Size and Characteristics	Intervention	Outcome
Does a resistance exercise session with continuous or intermittent blood flow restriction promote muscle damage and increase oxidative stress? Neto et al., 2018 [6].	Randomized-controlled trial	10 recreationally trained men	Continuous and intermittent low-intensity BFR vs. high-intensity BFR	High-intensity BFR increased creatine kinase levels, indicating greater muscle damage.
Blood flow restricted training leads to myocellular macrophage infiltration and upregulation of heat shock proteins, but no apparent muscle damage, Nielsen et al., 2017 [8].	Randomized-controlled trial	20 healthy men	3 weeks of training; 20% 1RM BFR, low (20% 1RM), and high (70% 1RM) resistance exercise	BFR training increased both pro-inflammatory (M1) and anti-inflammatory (M2) macrophages in skeletal muscle
Effects of blood flow restriction during moderate-intensity eccentric knee extensions, Behringer et al., 2018 [9].	Randomized-controlled trial	20 healthy male sports students	Moderate-intensity eccentric leg press with and without BFR	BFR exercises induced greater muscle swelling, metabolic stress, and acute increase in growth hormone.
Low-intensity resistance training with blood flow restriction improves vascular endothelial function and peripheral blood circulation in healthy elderly people, Shimizu et al., 2016 [7].	Randomized-controlled trial	40 healthy elderly people	Low-intensity resistance training with BFR	BFR increased plasma norepinephrine, lactate, vascular endothelial growth factor, and growth hormone production
Increases in a marker of immune system reconstitution are predated by decreases in 24-h urinary cortisol output and depressed mood during a 10-week stress management intervention in symptomatic HIV-infected men, Antoni et al., 2005 [11].	Randomized-controlled trial	25 men with HIV infection	10-week cognitive behavioral stress management; 6–12 months follow-up	Increased CD4 + CD45RA + CD29+ lymphocyte count
Stress management effects on psychological, endocrinological, and immune functioning in men with HIV infection: Empirical support for a psychoneuroimmunological model, Antoni et al., 2003 [10].	Narrative Review	HIV+ men	10-week group-based cognitive behavioral stress management	Increased CD4+ and CD8+ T cell subsets and IgG titers, reduced cortisol and norepinephrine
Acute increases in night-time plasma melatonin levels following a period of meditation, Tooley et al., 2000 [14].	Controlled crossover study	17 experienced meditators	1-h midnight meditation; Sidhi vs. yoga	Increased melatonin levels
Effect of rhythmic breathing (Sudarshan Kriya and Pranayam) on immune functions and tobacco addiction, Kochupillai et al., 2005 [13].	Controlled non-randomized trial	Cancer patients	Rhythmic Breathing (Sudarshan Kriya vs. Pranayam); 12–24 weeks follow-up	Increased NK cells, no change in T cells,
A structured psychiatric intervention for cancer patients: II. Changes over time in immunological measures, Fawzy et al., 1990 [12].	Randomized-controlled trial	61 Melanoma patients	6-week structured psychiatric group intervention; 6 months follow-up	Increased lymphocyte and NK cell cytotoxicity, decreased CD4+ T cell percentage
Impact of exercise in hypoxia on inflammatory cytokines in adults: A systematic review and meta-analysis, Khalafi et al., 2023 [15].	Meta-analysis	243 healthy adults	Hypoxic vs. normoxic exercise	IL-10 increase was greater in hypoxic conditions, TNF-α increased
The molecular adaptive responses of skeletal muscle to high-intensity exercise/training and hypoxia, Li et al., 2020 [19].	Narrative Review	Both human and animal studies	High intensity exercises in hypoxic environment	Anti-inflammatory cytokines increased; pro-inflammatory responses were inconsistent.
Vitamin E supplementation inhibits muscle damage and inflammation after moderate exercise in hypoxia, Santos et al., 2016 [16].	Randomized-controlled trial	16 healthy physically active men	Exercise under hypoxic vs. normoxic conditions with or without vitamin E supplementation	Lower post-exercise increases in IL-6 and TNF-α in hypoxic vs. normoxic conditions (not significant).
Carbohydrate and glutamine supplementation modulates the Th1/Th2 balance after exercise performed at a simulated altitude of 4500 m., Caris et al., 2014 [17].	Randomized-controlled trial	9 healthy physical active men	Three exercise sessions at 70% VO_2_peak until exhaustion under hypoxia	Increased IL-6 post-exercise and 2 h later, no change in TNF-α
Hypoxia-inducible factor-dependent induction of netrin-1 dampens inflammation caused by hypoxia, Rosenberg et al., 2009 [18].	Experimental study	Mice	Normobaric hypoxia (8% O2 environment)	Mucosal inflammation and increased circulating pro-inflammatory cytokines
Inflammatory cytokines response to isometric handgrip exercise and the effects of duration and intensity of the isometric effort in prehypertensive subjects, Ogbutor et al., 2022 [20].	Randomized-controlled trial	192 sedentary prehypertensive adults	Isometric handgrip exercise	Increased IL-10, decreased TNF-α and IL-6 levels
Immune system response to isometric handgrip exercise and effects of duration and intensity of the exercise protocol on selected immune system parameters in prehypertensives, Ogbutor et al., 2022 [21].	Randomized-controlled trial	192 sedentary prehypertensive adults	Isometric handgrip exercise	Increased CD4 cell count and CD4/CD8 T cell ratio, decreased CD8 cells
Comparison of the effects of total body resistance exercise and traditional resistance training on the immune system biomarker in inactive obese women. Beni & Bakhteyari, 2022 [23].	Quasi-experimental	28 sedentary obese women	Traditional vs. whole-body resistance training	No changes in neutrophil, lymphocyte, monocyte and eosinophil; only basophil differed between groups
Influence of isometric exercise combined with electromyostimulation on inflammatory cytokine levels, muscle strength, and knee joint function in elderly women with early knee osteoarthritis, Park et al., 2021 [22].	Randomized-controlled trial	75 elderly women with early knee osteoarthritis	8-week isometric exercise with/without whole-body electromyostimulation	Reduced IL-6, TNF-α, CRP, resistin; improved strength and function
Impact of aerobic exercise versus resisted exercise on endothelial activation markers and inflammatory cytokines among elderly, Abd El-Kader et al., 2019 [24].	Randomized-controlled trial	80 previously sedentary elderly subjects	6 months of aerobic vs. resistance exercise training	Increased IL-10; decreased ICAM-1, VCAM-1, E-selectin, TNF-α, and IL-6.
Inflammatory cytokines and immune system modulation by aerobic versus resisted exercise training for elderly, Abd El-Kader et al., 2018 [25].	Randomized-controlled trial	60 previously sedentary elderly subjects	6 months of aerobic vs. resistance exercise training	Increased CD3+, CD4+, CD8+ T cells and IL-10; decreased CD4/CD8 ratio, IL-6, and TNF-α.
Plasma inflammatory biomarkers response to aerobic versus resisted exercise training for chronic obstructive pulmonary disease patients, Abd El-Kader et al., 2016 [26].	Randomized-controlled trial	100 COPD patients	Aerobic exercise vs. resistance exercise training for 12 weeks	Decreased TNF-α, IL-2, IL-4, IL-6, and CRP; greater reduction with aerobic exercise.
Acute response of serum cortisol to different intensities of resisted exercise in the elderly, Taha & Mounir, 2019 [27].	Randomized-controlled trial	60 elderly patients	Low, moderate, and high-intensity resistance exercise	Low and moderate-intensity exercises reduced cortisol levels more effectively
Exercise reduces depression and inflammation but intensity matters, Paolucci et al., 2018 [28].	Randomized-controlled trial	61 university students	6 weeks of medium-intensity continuous exercise vs. HIIT	HIIT increased TNF-α and IL-6.
Neutrophil and monocyte bactericidal responses to 10 weeks of low-volume high-intensity interval or moderate-intensity continuous training in sedentary adults, Bartlett et al., 2017 [29].	Randomized-controlled trial	27 healthy sedentary adults	HIIT vs. moderate-intensity continuous exercise	Both exercises enhanced neutrophil and monocyte bactericidal responses

BFR: Blood flow restriction; RM: Repetition maximum; HIV: Human immunodeficiency virus; Ig: Immunoglobulin; NK: Natural killer; IL: Interleukin; TNF: Tumor necrosis factor; VO_2_peak: Peak oxygen uptake; ICAM-1: Intercellular adhesion molecule-1; VCAM-1: Vascular cell adhesion molecule-1; COPD: Chronic obstructive pulmonary disease; CRP: C-reactive protein; HIIT: High-intensity interval training.

## 4. Discussion

This narrative review synthesized evidence from 24 studies published between January 2000 and December 2024 to evaluate how different exercise modalities affect immune function. Across modalities, exercise influenced immune regulation through changes in cytokine production, immune cell subsets, innate immune activity, and stress-related hormones. While aerobic and mind–body interventions showed the most consistent anti-inflammatory and immunoprotective effects, modalities such as HIIT, BFR, and hypoxic training produced more variable outcomes, often reflecting protocol intensity, duration, and participant characteristics.

Several modalities, particularly aerobic exercise and mind–body interventions, were consistently associated with reductions in pro-inflammatory cytokines (IL-6, TNF-α) and increases in anti-inflammatory markers such as IL-10 [10,11,24,25,26]. Improvements in CD4/CD8 ratios and T cell subsets further suggest favorable adaptive immune modulation [20,21,24,25]. These changes may be mediated by downregulation of hypothalamic–pituitary–adrenal axis activity and improved immune regulation, aligning with findings from cancer and HIV populations showing enhanced lymphocyte activity and NK cell responses [10,11,12,13,14].

Mind–body practices and aerobic exercise were also linked to reductions in cortisol and norepinephrine [10,11,24,25,26], supporting their role in attenuating chronic stress signaling. By contrast, BFR and hypoxic training often triggered acute elevations in stress hormones, catecholamines, and growth factors [6,7,8,9,16,17,18,19]. Such responses may reflect transient physiological stress, potentially contributing to adaptation but also raising variability in reported immune effects.

HIIT and resistance-based protocols produced mixed effects. While HIIT induced transient elevations in IL-6 and TNF-α [28], it also enhanced neutrophil and monocyte bactericidal activity, reflecting improved innate immune surveillance [29]. Similarly, BFR training influenced macrophage polarization, promoting both pro-inflammatory (M1) and anti-inflammatory (M2) phenotypes [8]. These findings highlight that even when systemic cytokines indicate acute inflammation, certain exercise types may still strengthen first-line immune defenses.

Overall, aerobic and mind–body interventions emerged as the most reliable modalities for improving immune balance, particularly in older adults and individuals with chronic disease [10,11,12,13,14,24,25,26]. Isometric exercise showed generally favorable effects on inflammatory cytokines and T cell profiles, though results for total leukocyte counts were inconsistent [20,21,22,23]. In contrast, BFR, hypoxic training, and HIIT produced heterogeneous outcomes, with beneficial immune adaptations in some contexts but pro-inflammatory responses in others [6,7,8,9,15,16,17,18,19,28,29]. The magnitude and direction of these effects appeared strongly dependent on exercise intensity, duration, and study population.

The observed biomarker changes have potential clinical significance. Reductions in IL-6 and TNF-α may reflect decreased systemic inflammation, while increases in IL-10 and improvements in CD4/CD8 ratios suggest enhanced immune regulation. Enhanced neutrophil and monocyte function may indicate stronger innate immune defenses. These shifts could contribute to lowering chronic disease risk, improving recovery, or enhancing resilience to infections. Nevertheless, whether these immunological alterations translate into measurable health or performance benefits remains unclear, underscoring the need for longitudinal and mechanistic studies.

Several methodological limitations constrain the strength of current evidence. Most studies had small sample sizes, heterogeneous biomarker assays, and short follow-up periods, limiting generalizability. Exercise protocols also varied in intensity, frequency, and supervision, complicating direct comparisons. For some modalities, notably resistance training and HIIT, the number of eligible studies was very limited, weakening the robustness of conclusions. This review did not apply a formal risk of bias tool given its narrative design; however, potential methodological shortcomings of the included studies were explicitly considered when interpreting the findings.

Future research should prioritize adequately powered randomized trials with standardized exercise protocols and consistent immune outcome measures. Comparative studies that evaluate multiple modalities under controlled conditions are needed to clarify relative effects. Longer-term follow-up will help determine whether short-term immune changes translate into reductions in morbidity or performance gains. Including more diverse populations, such as women, older adults, and immunocompromised patients, would improve clinical relevance.

## 5. Conclusions

This narrative review synthesized evidence from 24 studies to evaluate how different exercise modalities influence immune responses. Overall, aerobic exercise and mind–body interventions emerged as the most reliable modalities for promoting immunoprotective effects, consistently associated with reduced IL-6 and TNF-α, increased IL-10 production, and improved T cell balance across a range of populations, including older adults and those with chronic disease. In contrast, HIIT, BFR, hypoxic, and isometric protocols demonstrated more heterogeneous effects, with benefits such as enhanced innate immune function or reduced stress hormone levels in some contexts, but also transient pro-inflammatory responses depending on intensity, duration, and participant characteristics. Evidence for isolated resistance training remains scarce, limiting definitive conclusions. These findings suggest that aerobic and mind–body exercises can be recommended as practical strategies to support immune health, particularly in vulnerable populations, whereas other modalities may be more suitable in specific contexts such as athletic training or controlled rehabilitation settings.

Future research should prioritize large, well-designed randomized trials with standardized protocols and mechanistic endpoints to clarify dose–response relationships and long-term clinical relevance. Such studies will be essential to determine whether observed biomarker changes translate into tangible outcomes, including reduced infection risk, improved recovery, or enhanced performance, thereby enabling more precise exercise prescriptions for immune modulation.

## Data Availability

No new data were created or analyzed in this study. The original contributions presented in this study are included in the article. Data sharing is not applicable to this article.

## Abbreviations

The following abbreviations are used in this manuscript:

BFR	Blood flow restriction
COPD	Chronic obstructive pulmonary disease
CRP	C-reactive protein
HIIT	High-intensity interval training
HIV	Human immunodeficiency virus
IL	Interleukin
Ig	Immunoglobulin
NK	Natural killer
RM	Repetition maximum
TNF	Tumor necrosis factor
VO_2_peak	Peak oxygen uptake
